# Transcatheter Edge-to-Edge Mitral Valve Repair for Severe Regurgitation in Cardiogenic Shock: A Comprehensive Review

**DOI:** 10.3390/jcdd12120455

**Published:** 2025-11-24

**Authors:** Medha Biswas, William Edward Katz, Matthew Suffoletto, Zachary Rhinehart, Anson Conrad Smith, Jeffrey Fowler, Leyla Elif Sade

**Affiliations:** 1 School of Medicine, University of Pittsburgh, Pittsburgh, PA 15213, USA; biswasm@upmc.edu (M.B.); katzwe@upmc.edu (W.E.K.); suffolettoms@upmc.edu (M.S.); rhinehartzj@upmc.edu (Z.R.); smithaj@upmc.edu (A.C.S.); fowlerja@upmc.edu (J.F.); 2 Department of Internal Medicine, Division of Cardiology, Heart and Vascular Institute, University of Pittsburgh Medical Center, Pittsburgh, PA 15213, USA

**Keywords:** M-TEER, MitraClip, cardiogenic shock, mitral regurgitation

## Abstract

Cardiogenic shock is a critical pathological state marked by end-organ hypoperfusion due to severe cardiac dysfunction and is associated with high mortality. A substantial portion of patients with cardiogenic shock have concomitant severe mitral regurgitation (MR), which exacerbates hemodynamic instability by reducing forward cardiac output and contributes to pulmonary edema and respiratory failure through regurgitant backflow. In this high-risk setting, mitral transcatheter edge-to-edge repair (M-TEER) offers a minimally invasive treatment that can lead to hemodynamic and symptomatic improvement and potential mortality benefit. Initially indicated for patients with severe MR at prohibitive surgical risk, M-TEER is now guideline-supported for both primary and secondary MR in select populations. Emerging data suggest that M-TEER can reduce heart failure hospitalizations and improve patient quality of life. As clinical indications for M-TEER continue to expand, there is growing interest in the role of M-TEER as a stabilizing intervention in patients with cardiogenic shock and severe MR. This review aims to synthesize the current evidence surrounding the use of M-TEER in cardiogenic shock with a focus on patient selection, procedural and clinical considerations, and short- and long-term outcomes.

## 1. Introduction

Mitral regurgitation (MR) is the most prevalent valvular heart disease [1]. With age, the incidence of MR increases, with an estimated prevalence of moderate or severe MR of over 10% in patients over the age of 75 [2].

MR can be broadly classified based on the underlying etiology into primary (degenerative) MR and secondary (functional) MR. Primary MR results from structural abnormalities of the mitral valve apparatus: leaflets, chordae tendineae, papillary muscles, or mitral annulus. In contrast, secondary MR is caused by left ventricular (LV) dysfunction or left atrial enlargement, leading to displacement of the papillary muscles resulting in leaflet tethering, annular dilatation, or decreased leaflet coaptation [3].

Before the advent of M-TEER, population studies show that only 15% of patients received surgical mitral valve intervention, with mitral valve repair favored over mitral valve replacement [1]. In the United States, there are two M-TEER systems available: the MitraClip System (Abbott Vascular, Santa Clara, CA, USA) and the PASCAL System (Edwards Lifesciences, Irvine, CA, USA). Initially approved for degenerative MR in patients at prohibitive surgical risk, M-TEER has become a valuable complement to guideline-directed medical therapy (GDMT) in patients with moderate-severe to severe functional MR, addressing an additional population in need. Clinical studies have shown that M-TEER leads to sustainable improvement in heart failure symptoms and reduction in all-cause mortality and heart failure hospitalization rates [4,5,6,7].

As the clinical application of M-TEER continues to expand, attention has been directed towards its potential therapeutic role in the acute setting of cardiogenic shock (CS). CS is characterized as a state of acute circulatory failure leading to end-organ hypoperfusion due to severe cardiac dysfunction. Despite advances in pharmacologic therapy and the use of mechanical circulatory support, refractory CS remains a highly fatal condition with in-hospital mortality rates approaching 50% [8]. Data show that 5% to 20% of hospitalizations for CS are complicated by significant MR. [9,10] Acute presentation or decompensation of MR can precipitate CS due to rapid hemodynamic deterioration. Alternatively, pre-existing MR may act as a bystander during an acute cardiovascular condition such as ischemia, further exacerbating circulatory compromise until cardiogenic shock ensues. These patients are frequently at prohibitive surgical risk with high Society of Thoracic Surgeons (STS) scores (>15%), low INTERMACS profiles, and dependence on temporary mechanical circulatory support and/or inotropes. In patients with severe MR contributing to hemodynamic instability, M-TEER may serve as an effective therapeutic option. This review will explore the pathophysiology of MR in CS, the role of M-TEER, considerations for patient selection and timing, benefits, risks, limitations, and controversies surrounding this emerging therapy.

## 2. Pathophysiology of Mitral Regurgitation in Cardiogenic Shock

In CS with concomitant MR, understanding the underlying pathology of MR can aid in determining the etiology of shock and how to approach therapeutic options. Acute MR resulting from acute chordal rupture, mitral leaflet perforation, and papillary muscle rupture can precipitate cardiogenic shock. Similarly, acute worsening of chronic MR in cases of ischemic MR or functional MR may exacerbate the hemodynamic compromise in cardiogenic shock. In other instances, severe MR may be a marker of advanced ventricular failure where correction of the MR would not improve the hemodynamic condition but may instead be detrimental due to afterload mismatch, making this arguably the most difficult scenario to identify with confidence. Transthoracic echocardiography (TTE) is useful when MR is suspected and may be sufficient to establish the etiology of MR. Transesophageal echocardiography (TEE) represents the method of choice to assess valve anatomy, leaflet quality, motion, and coaptation defect, to confirm MR severity, and to assess LV function, RV function, and dilatation, estimate pulmonary artery pressure, and visualize concomitant valvular diseases [11]. Yet, determining whether MR is the primary driver versus a bystander in CS remains challenging and is context dependent.

### 2.1. Primary (Degenerative) Mitral Regurgitation

The most common causes of MR include degenerative mitral valve disease, such as mitral valve prolapse and flail leaflet, as well as other etiologies such as infective endocarditis, rheumatic, toxic, and collagen vascular disease. Primary MR is a disease of the mitral valve apparatus (leaflets, chordae tendineae, papillary muscles, annulus) leading to mitral valve incompetence [12]. Valve incompetence over time can lead to volume overload of the left-sided cardiac chambers and can contribute to hemodynamic compromise, pulmonary edema, atrial fibrillation, and, in some situations, critical states such as cardiogenic shock. Patients with isolated degenerative MR have a lower comorbidity burden yet may present with acute pulmonary edema and cardiogenic shock secondary to chordal rupture. Since primary MR is due to a structural abnormality of the mitral valve apparatus, correction of the MR can restore acutely the hemodynamic deterioration and prevent irreversible changes [12]. However, hemodynamic instability requiring inotropes or mechanical circulatory support is rarely due to chronic degenerative MR.

### 2.2. Secondary (Functional) Mitral Regurgitation

The new understanding of functional MR suggests separating the classification into atrial functional MR vs. ventricular functional MR [13]. Atrial functional MR can result in significant MR from atrial enlargement, leading to mitral annular dilatation without significant LV dilatation [5]. In patients with atrial functional MR and concomitant atrial fibrillation, restoration of normal sinus rhythm and guideline-directed medical therapy (GDMT) can significantly improve mitral valve function and contribute to left atrial reverse remodeling [13]. Importantly, atrial functional MR is unlikely to be a driver of cardiogenic shock and may be treated without cardiac surgery when the left ventricle is not implicated [13]. Furthermore, M-TEER is challenging due to the planar coaptation of the leaflets and may be futile.

Ventricular functional (VF) MR refers to MR developing secondary to LV dysfunction in the absence of organic mitral valve disease [3]. VFMR carries a worse prognosis, likely in part due to the underlying cardiomyopathy, and the benefits of intervening on the regurgitant valve remain limited unless addressing the underlying LV pathology [3,4]. In VFMR, LV enlargement or dysfunction causes apical and lateral papillary muscle displacement and leaflet tethering, leading to a central regurgitant jet [3]. Severe VFMR directly increases the risk of death or the need for a heart transplant [3]. Major trials evaluating M-TEER in VFMR have yielded conflicting results, with the MITRA-FR clinical trial demonstrating no benefit, whereas the COAPT and RESHAPE-HF2 clinical trials reported significant benefit [6,14,15]. These conflicting outcomes are explained by population differences, as the MITRA-FR cohort exhibited larger ventricular volumes, suggesting a more advanced, irreversible LV remodeling state despite less severe MR as compared to the COAPT and RESHAPE-HF2 populations [6,14,15]. Patients with chronic LV remodeling and advanced ventricular dysfunction may have MR as a bystander, with less pronounced immediate hemodynamic benefit [3,12]. However, it is important to note that two large randomized clinical trials, COAPT and RESHAPE-HF2, have shown a significant reduction in the risk of heart failure hospitalization and improvements in the composite endpoint of all-cause mortality and health status [6,15]. The additional benefit of M-TEER may come from the ability to improve a patient’s hemodynamic state to allow for up-titration of guideline-directed medical therapy for LV reverse remodeling. In the randomized controlled MATTERHORN trial conducted among patients with heart failure and secondary MR, M-TEER was noninferior to mitral-valve surgery with respect to a composite of death, rehospitalization for heart failure, stroke, reintervention, or implantation of an assist device in the left ventricle at 1 year and was associated with a significantly lower major adverse event rate [16].

### 2.3. Ischemic Mitral Regurgitation

The SHOCK trial registry showed that about 10% of patients with acute MI-associated CS had severe MR [17]. Ischemic MR can be transient or fixed depending on the severity and chronicity of myocardial ischemia, ranging from reversible ischemia to irreversible infarction. Acute MI can result in papillary muscle or chordal rupture with a primary MR phenotype. Cardiogenic shock due to acute severe MR complicating acute MI is characterized by a predominant female gender, less ischemic ECG findings, more prevalent inferior and posterior MI, delayed presentation, and a low INTERMACS score, with 30% presenting in SCAI stage D or above [16]. Furthermore, 78.7% of the patients were shown to be inotrope dependent and 50.4% under mechanical circulatory support before TEER [9].

Acute ischemic MR is typically associated with disproportionate posterior leaflet tethering without changes in mitral annular diameter [18]. Unlike transient ischemic MR, fixed chronic ischemic MR is characterized by remodeling that affects both the LV geometry and mitral valve apparatus, including papillary muscle displacement, LV dysfunction, leaflet tethering, and annular dilatation [19,20]. Chronic ischemic MR is defined as MR occurring more than 16 days after an MI, accompanied by one or more LV segmental wall motion abnormalities and a structurally normal MV apparatus and normal chordae tendinea, reflecting its ventricular origin and therefore limited responsiveness to mitral valve intervention [21,22].

## 3. M-TEER

Urgent mitral valve surgery is lifesaving in acute hemodynamic decompensation due to severe MR. However, the surgical risk may be prohibitively high in the setting of cardiogenic shock. Modeled after the surgical Alfieri stitch, M-TEER provides a possible advantage over mitral valve surgery for selected patients at high surgical risk with severe MR. The EVEREST II trial, which enrolled patients regardless of MR type, showed that M-TEER was less effective at reducing MR compared to conventional surgery but showed superior safety and similar clinical improvements. At follow-up, M-TEER more often required surgery for residual MR within the first year, but rates between 1 and 5 years were comparable to surgical mitral valve intervention [7,23]. Subgroup analysis showed that there was less difference among patients over the age of 70 years and patients with functional MR [7,23]. Over time, there have been technical advances in the M-TEER MitraClip system, which is now on its 5th generation, and the advent of the PASCAL system, both of which demonstrated significant improvements in durable reduction in primary MR compared with earlier outcomes [24,25]. Building on these technical advances, ongoing prospective randomized clinical trials are investigating the safety and efficacy of M-TEER in patients with low to intermediate surgical risk, aiming to refine patient selection and clinical outcomes [26,27].

The 2020 ACC/AHA and 2025 ESC guidelines recommend mitral valve surgery (repair) over M-TEER as a class 1 indication for primary MR with symptoms or without symptoms and evidence of LVEF< 60% or end-systolic diameter > 40mm [12,28]. Apart from papillary muscle rupture, acute primary MR rarely results in cardiogenic shock and is generally best managed with surgical valve repair or replacement [28]. Both the 2020 ACC/AHA and 2025 ESC guidelines emphasize that in secondary VFMR, GDMT remains the first-line treatment [12,28]. The 2025 ESC guidelines report for patients with nonischemic severe VFMR who remain symptomatic despite optimization of GDMT, with or without cardiac resynchronization therapy, M-TEER is recommended as a class I intervention when the anatomy is suitable, to reduce HF hospitalizations and improve quality of life in hemodynamically stable patients with impaired LVEF, based on recent evidence [28]. In contrast, the 2020 AHA/ACC valvular heart disease guidelines list M-TEER as a class 2a recommendation for the same indication based on COAPT and RESHAPE-HF2 trials [12]. MV surgery is recommended in patients with severe VFMR if CABG is anticipated [10,28]. Yet, for patients with frailty, excess risk of mortality and morbidity who are deemed to be too high risk for surgical valve replacement and CABG, M-TEER, in addition to percutaneous coronary intervention, may offer a potential avenue to modify the disease process and is a class IIb recommendation in the 2025 ESC Guidelines for Valvular Heart Diseases [28]. However, no clear recommendation exists for hemodynamically unstable patients.

## 4. Patient Selection and Timing

In terms of timing, cardiogenic shock presents an urgency that requires expedited inpatient evaluation and rapid utilization of a multidisciplinary committee familiar with procedural considerations. With the promise of M-TEER to alter the downward trajectory seen in cardiogenic shock, choosing the correct patients is imperative. Degenerative MR characterized by flail or prolapse is generally more amenable to M-TEER than functional MR [29,30]. By analogy with a traffic light, there has been a proposed ‘green’, ‘yellow’, and ‘red’ light” terminology to categorize patients based on feasibility and chance of success with M-TEER [30]. Under the “green light” criteria, optimal valve morphology is defined by sufficient residual leaflet tissue, minimal risk of inducing clinically significant mitral stenosis, and absence of severe leaflet disruption or tears [29,30].

Patients at high risk of procedural failure, or “red light” patients, characterized by restricted leaflets as in Carpentier Class IIIa phenotype, radiation exposure, or other inflammatory conditions affecting the mitral valve, favor MTEER. Severe annular calcification restricting leaflet motion, prior surgical annuloplasty or ring, prohibitively small mitral valve area (<3.5 cm^2^) [29] are risk factors for mitral stenosis. Other anatomic factors noted to result in inadequate reduction in MR with M-TEER include perforation from endocarditis, active endocarditis, or conditions with extreme mitral valve complexity, such as Barlow’s disease, poor coaptation reserve, excessive tenting, or short/restricted posterior mitral leaflet (<5 mm) [29].

Apart from anatomic considerations, clinical and patient factors are also listed in the “red light” criteria. Clinical factors that limit the ability to perform M-TEER include intracardiac thrombus that is mobile, imaging limitations including the inability to perform a TEE and inadequate venous access or inability for trans-septal puncture which could be from caval interruption such as an IVC filter, a large ASD occluder device or the inability to gain enough height on transseptal approach to manipulate the delivery system to the valvular pathology [29]. Clinically, factors associated with futility in performing M-TEER included patients without expected meaningful 1-year survival, patients with less than moderate-severe MR, and patients with inotrope requirement not thought to be related to mitral valve disease [29].

## 5. M-TEER in Cardiogenic Shock: Benefits, Risks, Limitations, and Controversies

Given the promising trials showing M-TEER benefit, M-TEER procedures have been extended to additional indications. In that regard, assessment of the feasibility and safety of M-TEER in cardiogenic shock is of utmost importance. M-TEER has been increasingly utilized as a therapeutic option in cardiogenic shock with severe MR in the United States from 2014 to 2019 [10] (Table 1).

Although randomized-controlled studies on M-TEER in concomitant cardiogenic shock are challenging to conduct, valuable insight into the use of M-TEER in this setting can be obtained through retrospective analysis. A study of the Society of Thoracic Surgeons/American College of Cardiology Transcatheter Valve Therapy Registry from 2013 to 2021 showed that successful MR reduction was achievable in most patients with cardiogenic shock and was associated with significantly lower mortality and heart failure hospitalization at 1 year [31] (Table 1). Another multicenter retrospective study, including 471 patients, soon after acute MI with severe MR, reported that surgical mitral valve repair or replacement was associated with a higher mortality rate compared with M-TEER. In this specific study, 41% of the patients were in cardiogenic shock in the intervention group [32] (Table 1).

Case series and observational studies showed procedural success in 86–94% of patients, and 86–92% of patients had a reduction in MR from severe to less than 2 + post-TEER [33,35,36,37] (Table 1). In a study of 3797 patients with diagnosis coding of cardiogenic shock, inotrope use, or mechanical circulatory support, procedural success was achieved in 85.6% of patients. Patients had degenerative MR in 53.4% of cases and functional MR in 27.5% of cases, and device success was associated with significantly lower all-cause mortality and lower heart failure admission rates [31] (Table 1). Further supporting feasibility and benefit, an analysis of 596 propensity-matched CS patients that underwent M-TEER demonstrated a higher 30-day and 1-year survival despite longer ICU and hospital stays [37] (Table 1).

Hemodynamic data provide mechanistic insight into M-TEER’s benefit in CS. Hemodynamic improvement after M-TEER (weaning support, improved v-waves, improved forward flow) and better outcomes suggest that MR can be a major driver of the shock physiology, particularly in the setting of acute ischemia and acute MI [9,33,34,36]. However, sub-groups showed that the protective effect of M-TEER was not seen in groups requiring advanced mechanical circulatory support or patients on hemodialysis [10] (Table 1). A 30-day mortality rate was 20% in MI populations as compared to 13% in non-MI populations, whereas 1-year mortality was 14% versus 35%, respectively [38]. These findings favor MTEER more strongly in the setting of acute MI and suggest that the outcome benefit over the intermediate term may be less likely due to LV deterioration and progression of cardiovascular disease in the setting of chronic heart disease complicated by CS and severe MR. There is no data stating clearly what percentage of patients in cardiogenic shock had MR as the primary hemodynamic driver (versus MR being a contributing factor among many).

When successful, M-TEER has shown promise as a therapeutic option to improve mortality in cardiogenic shock with concomitant severe MR. Achieving a significant (>2-grade) reduction in MR severity is associated with improved short-term mortality [38]. In this complex scenario, the primary consideration is the anatomic feasibility of achieving a successful M-TEER procedure, followed by an assessment of the potential clinical benefit of M-TEER in the patient’s clinical context of cardiogenic shock physiology and comorbidities. Defining the mechanism of MR and characteristics of the valve using imaging (transthoracic echocardiography, transesophageal echocardiography, cardiac computerized tomography) is essential to addressing these questions, given the differing implications of MR etiology on the potential benefit of M-TEER. In conditions where the LV is irreversibly remodeled, M-TEER may be futile and not meaningfully improve the ongoing hemodynamic compromise [34]. Futility may be hemodynamically driven by worsening LV dysfunction with afterload mismatch after reducing the MR and forcing the stroke volume into the high impedance of the aorta with M-TEER [40].

**Figure 1:**
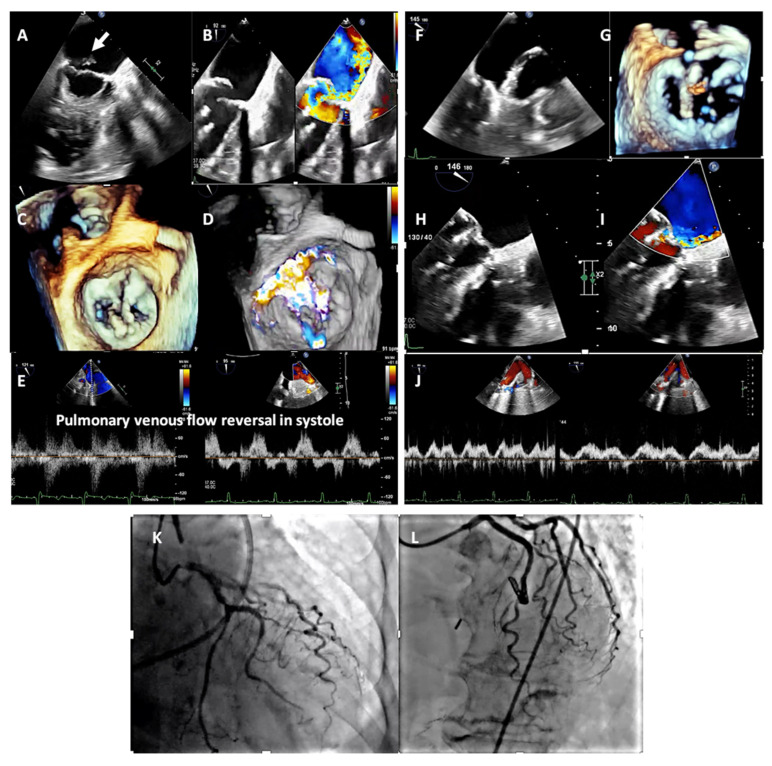
(**A**) 74-year-old woman who presented with acute onset chest pain and cardiogenic shock, hypoxemic respiratory, and multiorgan failure. She underwent emergent intubation, inotropic support, and an intra-aortic balloon pump. (**A**,**C**); papillary muscle rupture (arrow) on transesophageal echocardiogram (TEE). LV end-diastolic volume and EF were 65 mL/m^2^ and EF 65%, respectively. (**B**,**D**,**E**); findings of severe eccentric mitral regurgitation by 2D and 3D color Doppler and pulsed wave Doppler interrogation of pulmonary venous flow with systolic reversal, (**F**,**G**); TEE guided emergent TEER using 1 MitraClip XTW device, (**H**,**I**); immediate post M-TEER result with systolic blood pressure increasing to 130 from 90 mmHg and a decrease in left atrial pressure from 30 mm Hg to 15 mmHg, (**J**); restoration of normal pulmonary venous flow pattern, (**K**); Coronary angiography showing lesions in the left anterior descending and circumflex arteries in the acute phase, (**L**); result of coronary stenting 2 weeks after the hemodynamic stabilization with medical treatment and M-TEER.

Right ventricular (RV) dysfunction and dilatation should be considered a marker of chronic myocardial damage. When MR acutely drives hemodynamic compromise, imaging usually does not reveal severe left or right ventricular failure or dilation. Acute MR increases left atrial pressure and decreases forward flow. In this case, even with a driver of ischemia, intervention with M-TEER would acutely rescue the hemodynamic compromise in subjects deemed to have a prohibitive risk for surgery (Figure 1A–L, Videos S1A–E) as an adjunct to medical management and circulatory support. In the setting of active ischemia, prioritizing M-TEER over revascularization is a challenging multidisciplinary decision. Revascularization may be postponed until after M-TEER if the shock is primarily driven by acute MR amenable to correction with M-TEER.

In our experience, careful multidisciplinary discussion has been crucial in determining the role of M-TEER in cardiogenic shock with an emphasis on anatomic feasibility, ventricular remodeling, ongoing ischemia, and other clinical parameters to identify the likelihood of benefit from M-TEER. Aggressive therapies, including hemodynamic support with advanced circulatory assist devices, should be instituted without delay, while M-TEER can be considered as an adjunctive intervention and salvage therapy in cardiogenic shock.

Despite growing experience with M-TEER in cardiogenic shock, critical gaps remain, particularly regarding patient selection and the clinical characteristics that predict benefit [31,37]. In cardiogenic shock, severe MR increases LV end-diastolic pressure, creating a vicious cycle of forward and backward hemodynamic flow with increased vascular resistance that can rapidly worsen multiorgan dysfunction. While MR exacerbates LV volume overload, it paradoxically offloads the failing left ventricle. M-TEER reduces LV preload and potentially improves outcomes as seen in stable chronic heart failure with reduced ejection fraction. However, in patients with no ventricular contractile reserve, in patients requiring vasopressors or mechanical circulatory support, MR correction may increase the afterload and compromise LV function, making the benefit uncertain. Therefore, optimal timing and the benefit of M-TEER in this subset of patients remain debated. The futility of M-TEER due to afterload mismatch in advanced ventricular remodeling and the optimal timing of mechanical circulatory support require rigorous clinical trials [6,7,14,23,31,37]. To date, most retrospective registries are limited in inconsistent stratification of cardiogenic shock by SCAI stage and often categorize all cases of cardiogenic shock together without differentiation. As a result, based on existing data, it is not possible to determine how much benefit or futility can be expected from M-TEER in relation to the severity of CS. The negative effect of afterload mismatch could be particularly relevant in SCAI C and D, needing vasoconstriction and mechanical circulatory support.

Many studies are retrospective and included mixed populations without mechanistic separation (acute MI, chronic MR, functional vs. degenerative, non-ischemic etiologies). Controlled comparative studies assessing the impact of intervention on MR in cardiogenic shock are also lacking. The ongoing CAPITAL-MINOS trial (Transcatheter Mitral Valve Repair for Inotrope Dependent Cardiogenic Shock) that is evaluating the safety and efficacy of urgent M-TEER in inotrope-dependent CS aims to address some of the knowledge gaps to guide clinical practice [39].

## 6. Conclusions

M-TEER in the setting of cardiogenic shock is safe and feasible, with suggested improvement in 30-day and 1-year survival [17,27] (Table 1). The avoidance of cardiopulmonary bypass and a significantly lower complication profile make M-TEER an attractive emerging therapy in patients with cardiogenic shock and limited therapeutic options. M-TEER may serve as a salvage therapy in both functional and degenerative MR to reduce dependence on vasopressors and/or inotropes, and it can act as a bridge to long-term management, including cardiac transplantation. Given the encouraging results from observational and retrospective studies, randomized trials are underway [39]. The 2025 ESC guidelines now acknowledge the possible role of M-TEER in cardiogenic shock [28]. Standard definitions of shock severity and the mechanism of MR leading to CS should be aimed for future studies. Additional data is needed to define pathology-driven approaches and optimize the timing of M-TEER in cardiogenic shock. Furthermore, despite high procedural success rates, the durability of outcome benefit and its determinants are not completely answered. The key consideration is to identify severe MR as the main pathophysiologic driver of cardiogenic shock, with M-TEER beneficial only in patients with adequate left and right ventricular functional reserve and a reasonable life expectancy.

## Figures and Tables

**Table 1:** Summary of Current Evidence on M-TEER in Cardiogenic Shock.

Category	Study [Ref] (n)	Design/Population	Mean Age/% Male	Follow-Up Duration	Device Success	Key Findings
Studies	Jung et al. [9] (*n* = 141)	Multicenter, patient-level pooled analysis of TMVr in CS across 14 centers (patients with severe MR, hemodynamic instability, not surgical candidates).	68.9 ± 12.1 yr/55.3% male	30-day, 90-day, 1-year	88.7%	In-hospital mortality 15.6%, 90-day 29.5%, 1-year 42.6%. Procedural success independently predicted lower in-hospital and 90-day mortality (HR ≈ 0.36).
	Tang et al. [10] (*n* = 622)	Nationwide administrative (Medicare/NIS) analysis of CS hospitalizations; compared those receiving MitraClip vs. none.	71 ± 11 yr/58.4% male	In-hospital and 1-year outcomes	N/A	MitraClip is associated with lower in-hospital and 1-year mortality after matching.
	Simard et al. [31] (*n* = 3797)	STS/ACC TVT Registry analysis of TEER in CS with severe MR.	73.0 ± 11.9 yr/59.5% male	30-day and 1-year	85.6% (MR ≤ 2 + in 88.2%)	Successful MR reduction → lower 1-year mortality and HF hospitalizations.
	Haberman et al. [32] (*n* = 721)	Multicenter AMI cohort comparing medical, surgical, and percutaneous approaches (including CS subset).	73 ± 11 yr/57% male	Median ≈ 12 mo	Reported per group (>85%)	Percutaneous repair showed survival benefit vs. conservative therapy in post-AMI high-risk patients.
	Falasconi et al. (MITRA-SHOCK) [33] (*n* = 31)	Multicenter observational experience of TEER in refractory CS.	Median 73 (IQR 66–78); 74% male	30-day and 6-month	≈87%	30-day survival ≈78%; procedural success predicted survival.
	Droppa et al. [34] (*n* = 25)	Single-center hemodynamic study (pre/post TEER in CS).	65 yr/80% male	24–72 h and 30-day	100%	Demonstrated improved CO and LV filling pressures after TEER.
Meta-Analyses/Systematic Reviews	Saito et al. [35] (*n* = 4060)	Meta-analysis of observational studies of TEER in CS.	68.2 yr/58.6% male	Up to 1 year	≈90%	TEER reduced short-term mortality vs. conservative therapy; evidence quality limited.
	Ahmed et al. [36] (*n* pooled = 478)	Systematic review and meta-analysis focused on acute MR with CS.	Mean age ≈ 70 yr/62% male	30–90 days	≈92%	TEER is associated with lower in-hospital mortality compared with usual care; heterogeneity noted.
	Martinez-Gomez et al. [37] (*n* pooled ≈ 152)	Systematic review of MitraClip in hemodynamically unstable patients.	Mean age ≈ 69 yr/60% male	30-day and longer (limited)	≈89%	High procedural feasibility; limited randomized evidence.
	Dimitriadis et al. [38] (*n* pooled = 453)	Systematic review and meta-analysis of TEER in CS (pooled observational studies).	Pooled mean age ≈ 71 yr/63% male	30-day to 12 months	87–94%	Pooled 30-day survival ≈ 78%; M-TEER improved short-term outcomes vs. historical controls; heterogeneity limits certainty.
Ongoing Trials	Parlow et al. [39] CAPITAL MINOS (*n* planned 80)	Trial design: randomized M-TEER + medical therapy vs. medical therapy alone in inotrope-dependent CS.	Mean age ≈ 70 yr/N/A	30-day primary, 6-month secondary	N/A	Will assess survival free of MCS/transplant at 30 days; designed to provide randomized evidence for TEER in inotrope-dependent CS.

Major studies evaluating transcatheter mitral edge-to-edge repair (M-TEER) in patients with cardiogenic shock. Data include study type, patient population, procedural success, MR reduction, and clinical outcomes, including 30-day and 1-year mortality. Abbreviations: MR = mitral regurgitation; M-TEER = mitral transcatheter edge-to-edge repair; CS = cardiogenic shock; ICU = intensive care unit; HF = heart failure; LV = left ventricle; MI = myocardial infarction; N/A = not applicable; n = number.

## Data Availability

No new data were created for this article.

## Supplementary Materials

The following supporting information can be downloaded at: https://www.mdpi.com/article/10.3390/jcdd12120455/s1, Video S1A–E.

## Abbreviations

The following abbreviations are used in this manuscript:

GDMT	Guideline-directed medical therapy
LV	Left ventricle
MI	Myocardial infarction
M-TEER	Mitral transcatheter edge-to-edge repair
MR	Mitral regurgitation
VF	Ventricular functional
