# Supplementary material for: Transcatheter Edge-to-Edge Mitral Valve Repair for Severe Regurgitation in Cardiogenic Shock: A Comprehensive Review
Source: J Cardiovasc Dev Dis. 2025 Nov 24;12(12):455. doi: 10.3390/jcdd12120455 (PMC12734161; doi:10.3390/jcdd12120455)
Supplement: Supplementary file 1 [file jcdd-12-00455-s001.zip › jcdd-3905107-supplementary.pptx]

## Slide 1
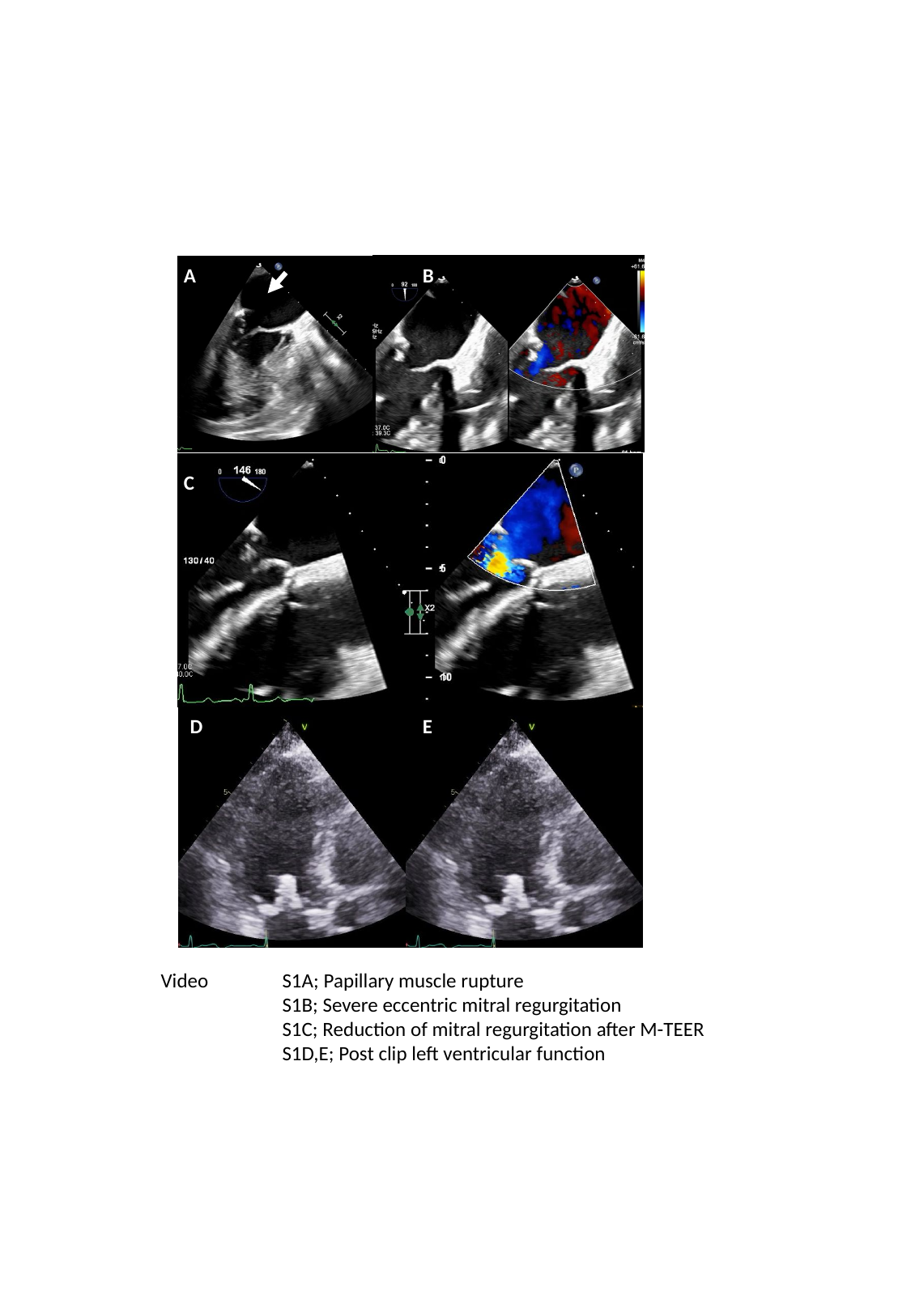

A
B
C
D
E
Video 	S1A; Papillary muscle rupture
	S1B; Severe eccentric mitral regurgitation
	S1C; Reduction of mitral regurgitation after M-TEER
	S1D,E; Post clip left ventricular function
